# Underestimation of Intraocular Pressure (IOP) After LASIK and PRK: Systematic Review and Meta-Analysis

**DOI:** 10.3390/jcm15124426

**Published:** 2026-06-08

**Authors:** Stamatios Lampsas, Efthymios Karmiris, George D. Kymionis, Irini Chatziralli

**Affiliations:** 1 2nd Department of Ophthalmology, Attikon University Hospital, Medical School, National and Kapodistrian University of Athens, 11528 Athens, Greece; tkarmiris@yahoo.com (E.K.); eirchat@yahoo.gr (I.C.); 2 1st Department of Ophthalmology, “G. Gennimatas” Hospital, Medical School, National and Kapodistrian University of Athens, 11528 Athens, Greece; gkymionis@gmail.com

**Keywords:** corneal biomechanics, intraocular pressure, LASIK, PRK, refractive surgery

## Abstract

**Background/Objectives**: Refractive corneal surgery alters corneal biomechanics and thickness, affecting the accuracy of intraocular pressure (IOP) measurements. This systematic review and meta-analysis aimed to quantify the postoperative underestimation of IOP following such procedures. **Methods**: This systematic review and meta-analysis, conducted based on PRISMA guidelines, evaluated pre- and postoperative IOP changes following LASIK and PRK using GAT, ORA, and CORVIS ST, based on studies identified through PubMed, Scopus, Cochrane Library, and ScienceDirect up to 31 December 2025, with results synthesized using random-effects models and reported as mean differences (MD) with 95% confidence intervals (CI). **Results**: Of the 1796 articles identified, 54 studies met the inclusion criteria, encompassing a total of 4730 eyes. After LASIK, a statistically significant underestimation of IOP was observed with all methods: GAT (MD: 3.23 mmHg, 95% CI: 2.77–3.69, *p* < 0.001), ORA (MD: 2.13 mmHg, 95% CI: 1.56–2.70, *p* < 0.001), and CORVIS ST (MD: 1.39 mmHg, 95% CI: 0.53–2.24, *p* = 0.001). Similarly, after PRK, a significant reduction in IOP was recorded with GAT (MD: 2.04 mmHg, 95% CI: 1.24–2.84, *p* < 0.001) and ORA (MD: 2.46 mmHg, 95% CI: 0.62–4.29, *p* < 0.01), while the difference measured by CORVIS ST was not statistically significant. **Conclusions**: LASIK and PRK result in systematic underestimation of IOP, most pronounced with GAT and less evident with ORA and CORVIS ST, highlighting the importance of selecting appropriate tonometry methods for accurate monitoring, especially in patients at risk of glaucoma or elevated IOP.

## 1. Introduction

Refractive errors (myopia, hyperopia, and presbyopia) is the number one cause of low visual acuity, accounting for approximately 51–58% of visual impairment [1,2]. Corneal refractive surgery, one of the most widely used techniques for correcting refractive errors of the eye, has been at the forefront of ophthalmologic interest over the past two decades due to significant advances in the field. In the United States, it is estimated that 700,000–800,000 LASIK procedures are performed annually, making it the most widely used corneal refractive surgical technique, while PRK accounts for approximately 10–15% of laser vision correction procedures, corresponding to roughly 70,000–120,000 surgeries per year [3].

Accurate measurement of IOP is a critical component of the ophthalmologic examination for the detection and monitoring of glaucoma, with Goldmann applanation tonometry (GAT) considered the gold standard among the various available tonometry methods [4]. Refractive corneal surgeries, such as Laser-Assisted In Situ Keratomileusis (LASIK) and Photorefractive Keratectomy (PRK), reduce central corneal thickness by removing tissue from the anterior stroma—the strongest layer—thereby altering biomechanical properties, decreasing overall stability, and weakening corneal rigidity, elasticity, and viscoelastic response [5,6]. The accuracy of intraocular pressure measurements may be significantly affected following these procedures due to alterations in the biomechanical properties of the cornea, thereby increasing the risk of underestimation and potentially resulting in delayed diagnosis of ocular hypertension and/or glaucoma [7,8]. Previous studies have shown that IOP is frequently underestimated after LASIK and PRK. Postoperative corneal thinning and alterations in corneal biomechanics reduce resistance during applanation tonometry, leading to lower measured IOP values. This underestimation may delay the detection and management of steroid-induced IOP elevation and has been associated with progression to advanced glaucoma [9,10]. However, contemporary devices such as the Ocular Response Analyzer (ORA) and Corneal Visualization Scheimpflug Technology (Corvis ST) enable more accurate estimation of IOP despite post-refractive alterations in corneal biomechanics, offering a comparative advantage over GAT [7,10,11,12]. Although these novel devices offer several advantages, the underestimation of IOP still exists and newer algorithms adapted both for Corvis ST and ORA have tried to improve this, with limited efficiency and accuracy [13]. Given the increasing number of patients undergoing refractive surgery worldwide and the critical role of accurate IOP assessment in glaucoma detection and monitoring, understanding the extent of postoperative IOP underestimation carries important clinical significance and was a major motivation for conducting this study.

Based on the above, the aim of this systematic review and meta-analysis is to evaluate the changes in IOP following corneal refractive surgeries in patients who have undergone LASIK and PRK. Secondarily, the study seeks to quantify these changes through meta-analysis, focusing on measurements obtained with GAT, the ORA and Corvis ST before and after surgery, as well as their relationship with alterations in corneal biomechanics.

## 2. Materials and Methods

### 2.1. Literature Search

This study followed the methodological approach specified by the Preferred Reporting Items for Systematic Reviews and Meta-Analyses (PRISMA) guidelines [14], and the protocol was officially recorded with PROSPERO under registration number CRD420261367793. The PRISMA 2020 checklist has been included in the Supplementary Materials (Table S1). The search queries were designed to apply to the endpoints of the protocol, investigating the changes of IOP before and after LASIK and PRK Refractive Surgery (Supplementary Table S2). Eligibility based on predefined protocol selection criteria was independently assessed by two researchers. Before conducting the final analysis, the database searches were rerun, and any newly identified relevant studies were obtained for inclusion. Additionally, the reference lists of the retrieved articles were also reviewed using the “snowballing” technique to identify obtain relevant publications.

### 2.2. Study Selection and Data Extraction

All primary observational research—including prospective and retrospective cohort studies, cross-sectional analyses, and case–control investigations—was incorporated based on the following selection criteria: (i) All included studies involved human adults aged 18 years or older, encompassing both male and female participants; (ii) Peer-reviewed articles published in English were considered for inclusion; (iii) Studies that assessed and measured IOP before and after refractive surgery—specifically PRK and/or LASIK—were included; (iv) IOP was measured using the GAT, the ORA, and/or the Corvis ST (v) Studies were included only if postoperative IOP was measured at one month after surgery, in order to avoid the influence of postoperative corticosteroids, which can elevate IOP. Only studies evaluating IOP using standard central corneal measurements were included to ensure consistency and comparability across the analyzed tonometry methods. Topical corticosteroid use in the eye can elevate intraocular pressure (IOP) by increasing aqueous outflow resistance due to the accumulation of extracellular matrix material within the trabecular meshwork, potentially leading to steroid-induced glaucoma [15]. The following were excluded: (i) all reviews, systematic reviews, and meta-analyses; (ii) studies not published in English; and (iii) animal studies. Additionally, the inclusion criteria were defined according to the PECOS framework to ensure transparency and accuracy in this systematic review and meta-analysis: (i) participants (P)-patients undergone PRK or LASIK; (ii) exposure (E)-patients undergone refractive surgery and IOP evaluated before surgery by GAT, or/and ORA, or/and Corvis ST; (iii) comparator (C)-patients undergone refractive surgery and IOP evaluated after surgery by GAT, or/and ORA, or/and Corvis ST; (iv) outcome (O)-mean difference of IOP before and after; (v) study (S)-observational studies. After the completion of study selection, two independent reviewers extracted the relevant data from the included studies utilizing a predefined data extraction form: (i) the name of the first author and the year of publication; (ii) the study sample-total eyes undergone refractive surgery; (iii) refractive surgery method (LASIK or/and PRK); (iv) the method of IOP evaluation; (v) mean age and male sex ratio for the whole sample. The screening process was performed after deduplication of citations using Rayyan systematic review software and Zotero (version 7.0.12).

### 2.3. Quality Assessment

To assess the methodological rigor and potential bias of the 54 studies that met the inclusion criteria, the Newcastle–Ottawa Scale (NOS)—modified appropriately for case-control, cross-sectional, and cohort designs—was employed. The NOS evaluation focuses on three key areas: (i) selection of participants, (ii) comparability of study groups, and (iii) ascertainment of exposure or outcome of interest. The maximum score is 9 for case-control studies and cohort studies, with scores of 0–3, 4–6, and 7–9 indicating low, moderate, and high quality, respectively. A detailed report is included in Supplementary Table S3.

### 2.4. Statistical Analysis

Forest plots were used to present the results, with group differences evaluated using mean differences (MDs) and corresponding 95% confidence intervals (CIs) calculated under a random-effects model. Statistical heterogeneity was assessed using the Q statistic based on the χ^2^ test (with a significance level of *p* = 0.05), while the I^2^ statistic quantified the proportion of variability attributable to between-study heterogeneity, with values >75% indicating substantial, 50–75% moderate, and <50% low heterogeneity. A sensitivity analysis was conducted using a leave-one-out approach to compare mean differences (MDs), while outlier studies were identified and excluded based on Egger’s test, a statistical method used to assess funnel plot asymmetry. Sensitivity analyses were not performed for meta-analyses including fewer than five studies, as such analyses are considered unreliable and may yield unstable estimates due to the limited number of included studies [16]. All statistical analyses were performed using RevMan 5.4 software (The Cochrane Collaboration, Oxford, UK).

## 3. Results

### 3.1. Search Results

**Figure 1:**
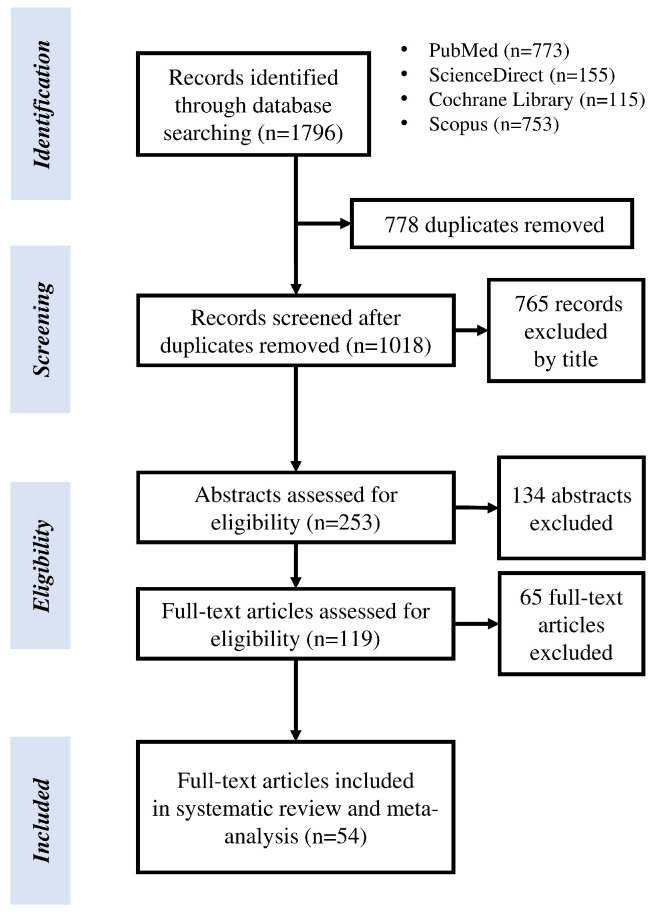
PRISMA flowchart for study selection.

A total of 54 studies were included in the systematic review and meta-analysis following the screening of 1796 initially identified records. Overall, 4730 eyes undergone refractive surgery were included, with a mean subject’s age of 32.7 ± 7.8 years, 45.3% of them being male, and a mean post refractive surgery follow-up time 4.3 months (Figure 1, Table 1).

### 3.2. Comparison of Pre- and Post-Operative IOP in Patients Who Have Undergone LASIK

#### 3.2.1. Measurement of Pre- and Post-Operative IOP Using a Goldmann Applanation Tonometer (GAT) After LASIK

**Figure 2:**
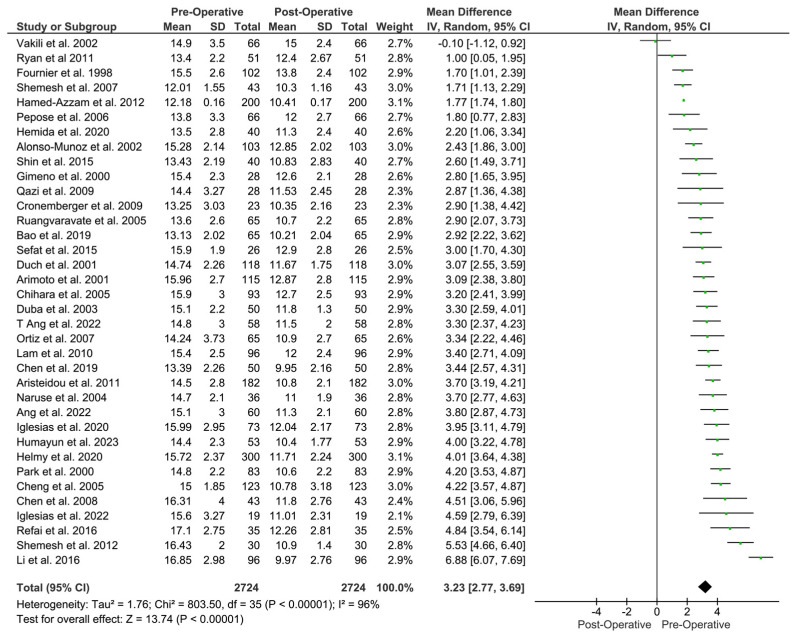
Forest plot of the estimated mean difference (MD) in intraocular pressure (IOP) measured with GAT before (pre-operative) and after (post-operative) LASIK. A random-effects model was applied; SD: standard deviation; CI: confidence interval; [8,21,23,25,26,29,32,33,34,35,36,37,38,39,40,41,42,43,45,46,47,48,51,52,53,54,55,56,57,58,59,60,63,65,66,67].

**Figure 3:**
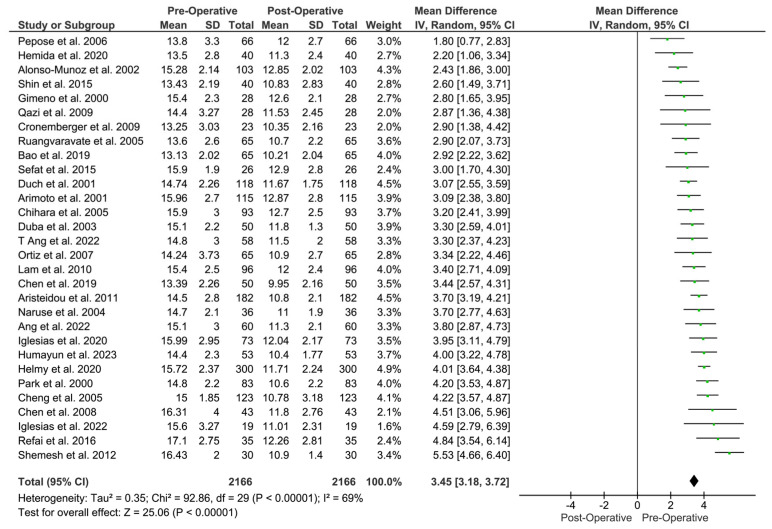
Forest plot of Sensitivity analysis with the estimated mean difference (MD) in intraocular pressure (IOP) measured with GAT before (pre-operative) and after (post-operative) LASIK. A random-effects model was applied; SD: standard deviation; CI: confidence interval; [8,21,23,25,26,29,32,33,35,36,37,38,39,40,42,43,45,46,48,51,52,55,56,57,58,60,63,65,66,67].

In the present analysis, 36 studies were included, examining a total of 2724 eyes before and after LASIK. This meta-analysis demonstrated a statistically significant difference in IOP measured with GAT, with a calculated mean difference of 3.23 mmHg (95% CI: 2.77–3.69, *p* < 0.001). Heterogeneity among the included studies was substantial, with I^2^ = 96% (Figure 2). In sensitivity analysis, a total of 30 studies were included, encompassing 2166 eyes evaluated before and after LASIK, six outlier studies were removed after funnel plot assessment. The findings of this analysis revealed a statistically significant decrease in IOP measured with GAT, with an estimated mean difference of 3.45 mmHg (95% CI: 3.18–3.72, *p* < 0.001). The level of heterogeneity across the included studies was moderate as indicated by an I^2^ value of 69% (Figure 3). Egger’s test did not indicate significant publication bias, as the intercept was not statistically significant (*p* = 0.495).

#### 3.2.2. Measurement of Pre- and Post-Operative IOP Using Ocular Response Analyzer (ORA) After LASIK

**Figure 4:**
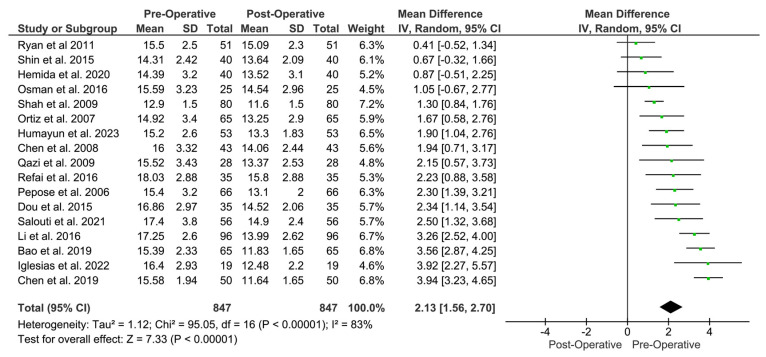
Forest plot of the estimated mean difference (MD) in intraocular pressure (IOP) measured with ORA before (pre-operative) and after (post-operative) LASIK. A random-effects model was applied; SD: standard deviation; CI: confidence interval; [8,11,23,25,26,30,31,32,33,34,38,39,47,48,51,58,64].

**Figure 5:**
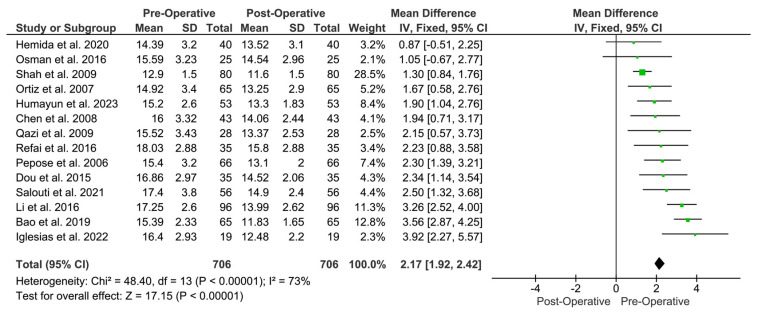
Forest plot of Sensitivity analysis with the estimated mean difference (MD) in intraocular pressure (IOP) measured with ORA before (pre-operative) and after (post-operative) LASIK. A random-effects model was applied; SD: standard deviation; CI: confidence interval; [8,11,25,26,30,31,32,33,39,47,48,51,58,64].

A total of 17 studies were included in this analysis, encompassing 847 eyes evaluated before and after LASIK. The meta-analysis revealed a statistically significant decrease in IOP measured with ORA, with an estimated mean difference of 2.13 mmHg (95% CI: 1.56–2.70, *p* < 0.001). Substantial heterogeneity was observed among the included studies, with an I^2^ value of 83% (Figure 4). In this analysis, 14 studies were included, comprising a total of 706 eyes evaluated before and after LASIK, three outlier studies were removed after funnel plot assessment. The meta-analysis demonstrated a statistically significant reduction in IOP measured with ORA, with a calculated mean difference of 2.17 mmHg (95% CI: 1.92–2.42, *p* < 0.001). The heterogeneity among the included studies was moderate, with an I^2^ value of 73% (Figure 5). No evidence of publication bias was detected by Egger’s test, as the intercept did not reach statistical significance (*p* = 0.649).

#### 3.2.3. Measurement of Pre- and Post-Operative IOP Using Corneal Visualization Scheimpflug Technology (Corvis ST) After LASIK

**Figure 6:**
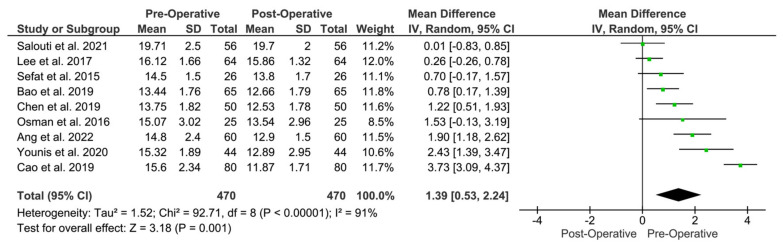
Forest plot of the estimated mean difference (MD) in intraocular pressure (IOP) measured with Corvis ST before (pre-operative) and after (post-operative) LASIK. A random-effects model was applied; SD: standard deviation; CI: confidence interval; [8,11,17,24,27,31,38,40,43].

**Figure 7:**
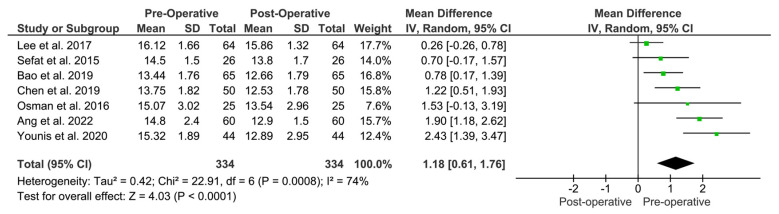
Forest plot of Sensitivity analysis with the estimated mean difference (MD) in intraocular pressure (IOP) measured with Corvis ST before (pre-operative) and after (post-operative) LASIK. A random-effects model was applied; SD: standard deviation; CI: confidence interval; [8,17,24,31,38,40,43].

A total of 9 studies were included in this analysis, encompassing 470 eyes assessed before and after LASIK. The meta-analysis revealed a statistically significant decrease in IOP measured with Corvis ST, with an estimated mean difference of 1.39 mmHg (95% CI: 0.53–2.24, *p* = 0.001). Substantial heterogeneity was observed among the included studies, with an I^2^ value of 91% (Figure 6). A total of 7 studies were incorporated into the sensitivity analysis, after two outlier studies removed, including 334 eyes evaluated before and after LASIK. The meta-analysis indicated a statistically significant reduction IOP measured with Corvis ST, with an estimated mean difference of 1.18 mmHg (95% CI: 0.61–1.76, *p* < 0.0001). Moderate heterogeneity was identified among the included studies (I^2^ = 74%). Egger’s regression analysis was performed to assess potential publication bias and did not reveal statistically significant small-study effects (intercept = 3.61, 95% CI: −2.01 to 9.23, *p* = 0.159), suggesting no evidence of publication bias (Figure 7).

### 3.3. Comparison of Pre- and Post-Operative IOP in Patients Who Have Undergone PRK

#### 3.3.1. Measurement of Pre- and Post-Operative IOP Using GAT After PRK

**Figure 8:**
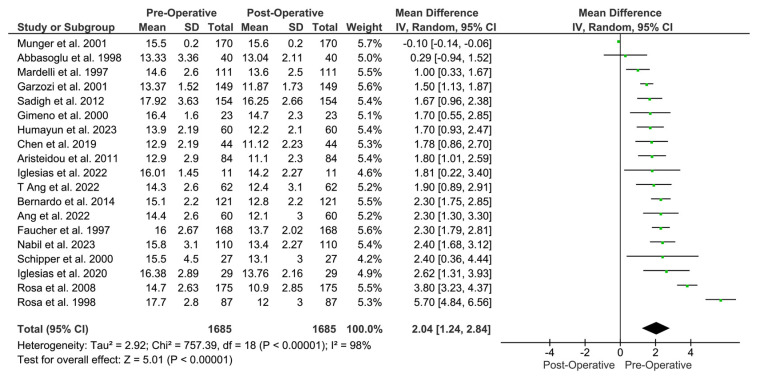
Forest plot of the estimated mean difference (MD) in intraocular pressure (IOP) measured with GAT before (pre-operative) and after (post-operative) PRK. A random-effects model was applied; SD: standard deviation; CI: confidence interval; [18,19,20,22,28,29,38,43,45,48,49,50,51,52,53,57,61,62].

**Figure 9:**
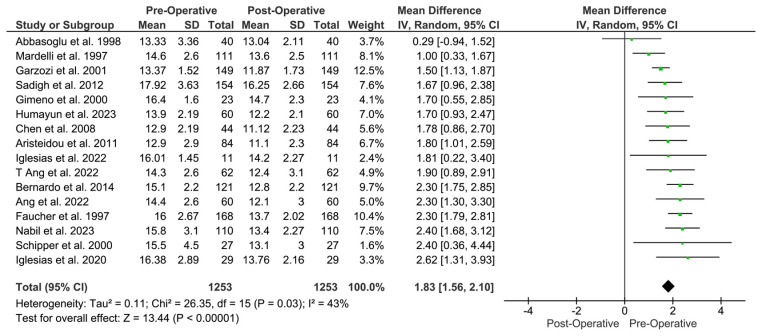
Forest plot of Sensitivity analysis with the estimated mean difference (MD) in intraocular pressure (IOP) measured with GAT before (pre-operative) and after (post-operative) PRK. A random-effects model was applied; SD: standard deviation; CI: confidence interval; [18,19,22,29,32,43,45,48,49,50,51,53,57,61,62].

In this analysis, 19 studies were included, comprising a total of 1685 eyes evaluated before and after PRK. The meta-analysis demonstrated a statistically significant reduction in IOP measured with GAT, with a calculated mean difference of 2.04 mmHg (95% CI: 1.24–2.84, *p* < 0.00001). The heterogeneity among the included studies was substantial, with an I^2^ value of 98% (Figure 8). A sensitivity analysis was performed by excluding three studies after funnel plot evaluation, resulting in 16 studies comprising 1253 eyes evaluated before and after PRK. The analysis continued to demonstrate a statistically significant reduction in IOP measured with GAT, with a mean difference of 1.83 mmHg (95% CI: 1.56–2.10, *p* < 0.00001). Notably, heterogeneity was low compared to the primary analysis, with an I^2^ value of 43%, indicating improved consistency among the included studies (Figure 9). Egger’s test did not demonstrate significant publication bias (intercept = 0.22, *p* = 0.804).

#### 3.3.2. Measurement of Pre- and Post-Operative IOP Using Ocular Response Analyzer (ORA) After PRK

**Figure 10:**
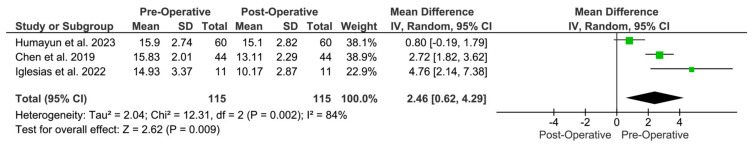
Forest plot of the estimated mean difference (MD) in intraocular pressure (IOP) measured with ORA before (pre-operative) and after (post-operative) PRK. A random-effects model was applied; SD: standard deviation; CI: confidence interval; [38,48,51].

A total of 3 studies were included in this analysis, encompassing 115 eyes assessed before and after PRK. The meta-analysis indicated a statistically significant decrease in IOP measured with ORA, with an estimated mean difference of 2.46 mmHg (95% CI: 0.62–4.29, *p* = 0.009). Substantial heterogeneity was observed among the included studies, with an I^2^ value of 84% (Figure 10).

#### 3.3.3. Measurement of Pre- and Post-Operative IOP Using Corneal Visualization Scheimpflug Technology (Corvis ST) After PRK

**Figure 11:**
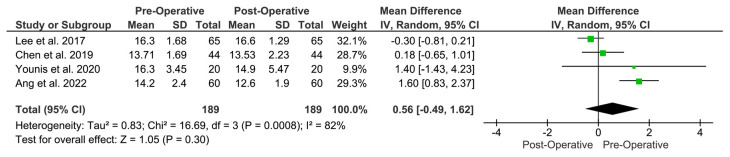
Forest plot of the estimated mean difference (MD) in intraocular pressure (IOP) measured with Corvis ST before (pre-operative) and after (post-operative) PRK. A random-effects model was applied; SD: standard deviation; CI: confidence interval; [17,24,38,43].

In this analysis, 4 studies were included, comprising a total of 189 eyes evaluated before and after PRK. The meta-analysis did not demonstrate a statistically significant difference in IOP measured with Corvis ST, with a calculated mean difference of 0.56 mmHg (95% CI: −0.49 to 1.62, *p* = 0.30). The heterogeneity among the included studies was considerable, with an I^2^ value of 82% (Figure 11). 

## 4. Discussion

This systematic review and meta-analysis comprehensively evaluated the impact of refractive procedures, namely LASIK and PRK, on IOP measurements using three different tonometry technologies: GAT, ORA, and CORVIS ST. The findings suggest that both LASIK and PRK result in a statistically significant postoperative underestimation of IOP, with the magnitude of underestimation being smaller when measured with CORVIS ST, which, through Scheimpflug imaging, appears to underestimate IOP to a lesser extent.

Specifically, following LASIK, the greatest underestimation of IOP was observed with GAT, with a mean reduction of 3.23 mmHg, followed by ORA with a decrease of 2.13 mmHg and CORVIS ST with 1.39 mmHg. LASIK induces thinning of the central cornea and disruption of stromal architecture, resulting in increased corneal deformability under the force applied during IOP measurement [23]. This increased deformability reduces the force required to applanate the cornea, with the magnitude of underestimation being proportional to the reduction in corneal hysteresis and corneal resistance factor, as well as to the amount of tissue ablated and the final central corneal thickness [68]. These meta-analytic findings further confirm that GAT underestimates IOP to a greater extent, as its measurements are based on the degree of corneal applanation [69].

Similarly, in patients undergoing PRK, a statistically significant reduction in measured IOP was observed with GAT and ORA, with mean differences of 2.04 mmHg and 2.46 mmHg, respectively. Moreover, sensitivity analysis for GAT measurements after PRK demonstrated that the pooled effect estimates remained stable after exclusion of outlier studies, supporting the robustness of the observed postoperative IOP reduction despite the initial heterogeneity. In contrast, IOP measurements obtained with CORVIS ST after PRK did not demonstrate a statistically significant change, suggesting that this technology may be more resistant to corneal alterations induced by the procedure. However, these findings should be interpreted cautiously, as the analysis of Corvis ST after PRK included only a limited number of studies and eyes, reducing statistical power and increasing the possibility of a Type II error. Although PRK does not involve the creation of a corneal flap, changes in corneal structure and biomechanical properties appear sufficient to affect IOP measurements. Biomechanical simulation studies further indicate that LASIK alters corneal stress distribution, reducing its resistance to deformation and thereby increasing measurement error [70,71]. This effect is more pronounced with deeper tissue ablation and thicker flaps and is more evident in LASIK compared to surface ablation techniques such as PRK.

With regard to corneal biomechanical properties, accounting for their alterations may improve the accuracy of postoperative IOP assessment, with corneal hysteresis representing a key parameter for quantifying these changes. The cornea exhibits viscoelastic behaviour, meaning it possesses both viscous and elastic properties, similar to most biological tissues. Viscoelastic materials demonstrate a degree of hysteresis during deformation, reflecting their capacity to dissipate energy when subjected to external forces [72]. Corneal hysteresis represents the ability of corneal tissue to absorb and release energy during the process of bidirectional applanation and constitutes an important biomechanical property of the cornea [72]. It reflects the cornea’s capacity to dampen mechanical stress, the balance between its elastic and viscous components, and the overall structural integrity of the tissue [73]. Clinically, reduced corneal hysteresis has been associated with glaucoma progression, provides insight into corneal biomechanical stability and the risk of postoperative ectasia following LASIK, and is commonly decreased in keratoconus, contributing to the diagnosis and monitoring of the disease [74,75].

Similarly, the corneal resistance factor (CRF) is a parameter measured by ORA that reflects the overall resistance of the cornea to deformation, incorporating its viscoelastic properties [68,76]. It is strongly correlated with central corneal thickness and overall corneal stiffness and is derived from the difference between inward and outward applanation pressures during air-puff–induced deformation [77,78]. Following refractive procedures such as LASIK and PRK, both CRF and corneal hysteresis are significantly reduced, with a more pronounced decrease observed after LASIK compared to PRK, reflecting greater biomechanical weakening due to flap creation and deeper stromal ablation [79,80,81]. Moreover, recent evidence demonstrates that LASIK produces significantly greater reductions in both CH and CRF compared to PRK, reflecting additional biomechanical weakening from flap creation and deeper stromal ablation [82]. Finite element simulations confirm that LASIK concentrates stress in the posterior stroma while PRK maintains more uniform anterior stress distribution, directly explaining the greater IOP underestimation observed after LASIK. Newer technologies offer only partial correction, as a 2024 study found that Corvis ST’s bIOP remained stable after PRK (change: 0.3 ± 1.7 mmHg) while ORA’s IOPcc still decreased significantly (−1.6 ± 4.0 mmHg), and the two devices should not be used interchangeably [12]. Thus, integrating CH and CRF as central interpretive variables provides a more complete framework for understanding why even advanced tonometers offer only partial correction of post-refractive IOP measurement error.

Finally, beyond the multifactorial nature of IOP measurement discussed above, a deeper understanding of how each tonometer’s operating principle influences post-refractive readings is essential for interpreting the observed differences between GAT, ORA, and Corvis ST. GAT measures IOP based on the force required to applanate a fixed corneal area, operating under the Imbert-Fick law that assumes an ideal spherical, thin, dry, and elastic cornea [83]. Refractive surgery violates these assumptions through corneal thinning, flattening, and altered biomechanics [84]. Consequently, GAT predominantly measures measurement-related bias rather than true IOP, as reduced thickness and increased deformability lower applanation force without any actual change in true IOP [84]. In contrast, ORA partly adjusts for biomechanical changes by assessing both inward and outward applanation pressures and deriving corneal hysteresis and corneal resistance factors, thereby providing a corneal-compensated IOP that may more accurately reflect true IOP compared with GAT [12]. Corvis ST further enhances assessment by employing an ultra-high-speed Scheimpflug imaging system to monitor dynamic corneal deformation, allowing estimation of a biomechanically adjusted IOP that incorporates individual differences in corneal stiffness and thickness through biomechanical modeling [85]. Thus, these devices differ in accuracy, with GAT showing the greatest bias, Corvis ST the least, and ORA demonstrating intermediate performance. Postoperative IOP measurements should therefore not be considered interchangeable.

A limitation of this meta-analysis is the substantial heterogeneity observed across several analyses, likely due to differences in surgical techniques, corneal characteristics, and follow-up duration among studies. Additionally, subgroup analyses and meta-regression were limited by the inconsistent reporting of variables such as ablation depth and central corneal thickness. Furthermore, differences in postoperative follow-up duration may have contributed to the observed heterogeneity, as IOP measurements can vary over time after refractive surgery (Supplementary Table S4). Another limitation of this meta-analysis is that physiological variability in IOP, including circadian fluctuations, body position, and recent diagnostic or therapeutic interventions, was not consistently reported across the included studies [86]. These factors may influence IOP measurements and could contribute to variability in postoperative assessments, particularly in post-refractive surgery patients where measurement accuracy is already affected by corneal alterations.

Clinically, the findings of this meta-analysis are particularly relevant for patients with a history of refractive surgery who are being monitored for glaucoma or elevated intraocular pressure. However, the high heterogeneity observed across most analyses suggests that the results should be interpreted with caution. In glaucoma suspects, clinicians must integrate other diagnostic tools, including optic nerve evaluation, retinal nerve fiber layer imaging, and visual field testing, rather than relying solely on measured IOP values. Finally, despite the development of newer technologies providing biomechanically adjusted IOP measurements, such as ORA and Corvis ST, clinicians should remain aware of the potential underestimation of IOP after refractive surgery and perform a comprehensive ophthalmologic evaluation.

## 5. Conclusions

This systematic review and meta-analysis comprehensively evaluated the impact of refractive procedures, namely LASIK and PRK, on IOP measurements using three different tonometry technologies: GAT, ORA, and CORVIS ST. The findings indicate that both LASIK and PRK lead to a statistically significant postoperative underestimation of IOP, with the magnitude showing differences among tonometry methods. Clinicians should be aware that even biomechanically adjusted IOP readings may underestimate true IOP post refractive surgery, warranting caution in glaucoma screening and monitoring.

## Figures and Tables

**Table 1:** Characteristics and findings of the included studies.

Study	Eyes	Refractive Method	IOP Measurement	Male Sex (%)	Age (Yes)
Younis et al., 2020 [17]	64	LASIK, PRK	CORVIS ST	66	26.3 ± 5
Sadigh et al., 2012 [18]	154	PRK	GAT	80.5	27.5 ± 6.3
Faucher et al., 1997 [19]	168	PRK	GAT	41.6	37.7 ± 9.3
Rosa et al., 2008 [20]	175	PRK	GAT	40	32 ± 8.7
Salouti et al., 2021 [11]	56	LASIK	CORVIS ST, ORA	25	29.1 ± 6.3
Alonso-Munoz et al., 2002 [21]	103	LASIK	GAT	48.7	30.6 ± 7.6
Bao et al., 2019 [8]	65	LASIK	CORVIS ST, ORA, GAT	53.3	26.6 ± 6.6
Schipper et al., 2000 [22]	27	PRK	GAT	NA	37.2 ± 9.5
Shin et al., 2015 [23]	40	LASIK	ORA, GAT	33.3	26.2 ± 7.2
Lee et al., 2017 [24]	64	LASIK, PRK	CORVIS ST	27.7	28.1 ± 5.4
Hemida et al., 2020 [25]	40	LASIK	ORA, GAT	NA	25.3 ± 3.7
Pepose et al., 2006 [26]	66	LASIK	ORA, GAT	42.4	39.6 ± 11.4
Cao et al., 2019 [27]	80	LASIK	CORVIS ST	45	25.3 ± 5.1
Munger et al., 2001 [28]	170	PRK	GAT	NA	51.8 ± 7.8
Aristeidou et al., 2011 [29]	266	LASIK, PRK	GAT	31.2	31.1 ± 10.2
Dou et al., 2015 [30]	35	LASIK	ORA	40	23 ± 3.4
Ang et al., 2022 [10]	60	LASIK, PRK	CORVIS ST, GAT	32.5	29.9 ± 7.9
Osman et al., 2016 [31]	25	LASIK	CORVIS ST, ORA	48	26.2 ± 3.4
Chen et al., 2008 [32]	43	LASIK	ORA, GAT	NA	40.5 ± 10.4
Ortiz et al., 2007 [33]	65	LASIK	ORA, GAT	53.8	37 ± 3.8
Ryan et al., 2011 [34]	51	LASIK	ORA, GAT	58	36 ± 8
Duba et al., 2003 [35]	50	LASIK	GAT	38	35 ± 4
Lam et al., 2010 [36]	96	LASIK	GAT	22.9	30.7 ± 6.7
Vakili et al., 2002 [37]	66	LASIK	GAT	44.1	39.0 ± 8.4
Chen et al., 2019 [38]	50	LASIK, PRK	CORVIS ST, ORA, GAT	38.2	26.3 ± 5.2
Refai et al., 2016 [39]	35	LASIK	ORA, GAT	73.6	30.1 ±7.1
Sefat et al., 2015 [40]	26	LASIK	CORVIS ST, GAT	60	36.6 ± 7.4
Hamed-Azzam et al., 2012 [41]	200	LASIK	GAT	23.5	31.9 ± 9.8
Naruse et al., 2004 [42]	36	LASIK	GAT	31.8	32.6 ± 9
T Ang et al., 2022 [43]	120	LASIK, PRK	GAT	40.8	30 ± 8
Rosa et al., 1998 [44]	87	PRK	GAT	52.8	27.5 ± 4.3
Gimeno et al., 2000 [45]	51	LASIK, PRK	GAT	46	29.7 ± 5.3
Helmy et al., 2020 [46]	300	LASIK	GAT	45	34.7 ± 8.8
Li et al., 2016 [47]	96	LASIK	ORA, GAT	42.7	24 ± 6
Humayun et al., 2023 [48]	113	LASIK, PRK	ORA, GAT	51.3	23.9 ± 5.2
Bernardo et al., 2014 [49]	121	PRK	GAT	50	34 ± 9
Nabil et al., 2023 [50]	110	PRK	GAT	55.8	28.2 ± 6.2
Iglesias et al., 2022 [51]	30	LASIK, PRK	ORA, GAT	NA	30.7 ± 6.5
Cronemberger et al., 2009 [52]	23	LASIK	GAT	32	37.8 ± 6.1
Garzozi et al., 2001 [53]	149	PRK	GAT	44.3	31.1 ± 8.2
Fournier et al., 1998 [54]	102	LASIK	GAT	40.6	38.7 ± 10
Cheng et al., 2005 [55]	123	LASIK	GAT	NA	33.8 ± 6.1
Shemesh et al., 2012 [56]	51	LASIK	GAT	50.9	26.6 ± 5.8
Iglesias et al., 2020 [57]	102	LASIK, PRK	GAT	61.8	31.6 ± 6.1
Qazi et al., 2009 [58]	28	LASIK	ORA, GAT	42.8	39 ± 12
Shemesh et al., 2007 [59]	43	LASIK	GAT	41.8	31.4 ± 9.7
Park et al., 2000 [60]	83	LASIK	GAT	NA	29.5 ± 7.3
Abbasoglu et al., 1998 [61]	40	PRK	GAT	33.3	42 ± 12
Mardelli et al., 1997 [62]	111	PRK	GAT	56.7	37.9 ± 9.2
Chihara et al., 2005 [63]	93	LASIK	GAT	54.8	33.2 ± 8.6
Shah et al., 2009 [64]	80	LASIK	ORA	39	41.2 ± 10.8
Ruangvaravate et al., 2005 [65]	65	LASIK	GAT	47.7	30.9 ± 7.1
Duch et al., 2001 [66]	118	LASIK	GAT	38.3	36.7 ± 7.6
Arimoto et al., 2001 [67]	115	LASIK	GAT	72.3	31.2 ± 10.5

LASIK, Laser-Assisted In Situ Keratomileusis; PRK, Photorefractive Keratectomy; IOP, Intraocular Pressure; GAT, Goldmann Applanation Tonometry; CORVIS ST, Corneal Visualization Scheimpflug Technology.

## Data Availability

All data generated or analyzed during this study are included in this article. Further enquiries can be directed to the corresponding author.

## Supplementary Materials

The following supporting information can be downloaded at: https://www.mdpi.com/article/10.3390/jcm15124426/s1, Supplementary Table S1: PRISMA 2020 Checklist. Supplementary Table S2: Search queries regarding the changes of Intraocular pressure before and after Laser-Assisted In Situ Keratomileusis (LASIK) and Photorefractive Keratectomy (PRK) Refractive Surgery; Supplementary Table S3: Quality assessment results obtained using the Newcastle–Ottawa Quality Assessment Scale (NOS) tool; Supplementary Table S4: Baseline corneal characteristics of the included studies.
