# Supplementary material for: Underestimation of Intraocular Pressure (IOP) After LASIK and PRK: Systematic Review and Meta-Analysis
Source: J Clin Med. 2026 Jun 8;15(12):4426. doi: 10.3390/jcm15124426 (PMC13300807; doi:10.3390/jcm15124426)
Supplement: Supplementary file 1 [file jcm-15-04426-s001.zip › jcm-4287306-supplementary.pdf]

Systematic Review

Underestimation of Intraocular Pressure (IOP) after LASIK and PRK: Systematic Review and Meta-analysis

Supplementary Material

Supplementary Table S1: PRISMA 2020 Checklist

Section and Topic	Item #	Checklist item	Location where item is reported
**TITLE**			
Title	1	Identify the report as a systematic review.	1
**ABSTRACT**			
Abstract	2	See the PRISMA 2020 for Abstracts checklist.	1
**INTRODUCTION**			
Rationale	3	Describe the rationale for the review in the context of existing knowledge.	1-2
Objectives	4	Provide an explicit statement of the objective(s) or question(s) the review addresses.	1-2
**METHODS**			
Eligibility criteria	5	Specify the inclusion and exclusion criteria for the review and how studies were grouped for the syntheses.	2-3
Information sources	6	Specify all databases, registers, websites, organisations, reference lists and other sources searched or consulted to identify studies. Specify the date when each source was last searched or consulted.	2-3
Search strategy	7	Present the full search strategies for all databases, registers and websites, including any filters and limits used.	2-3
Selection process	8	Specify the methods used to decide whether a study met the inclusion criteria of the review, including how many reviewers screened each record and each report retrieved, whether they worked independently, and if applicable, details of automation tools used in the process.	2-3
Data collection process	9	Specify the methods used to collect data from reports, including how many reviewers collected data from each report, whether they worked independently, any processes for obtaining or confirming data from study investigators, and if applicable, details of automation tools used in the process.	2-3
Data items	10a	List and define all outcomes for which data were sought. Specify whether all results that were compatible with each outcome domain in each study were sought (e.g. for all measures, time points, analyses), and if not, the methods used to decide which results to collect.	2-3
	10b	List and define all other variables for which data were sought (e.g. participant and intervention characteristics, funding sources). Describe any assumptions made about any missing or unclear information.	2-3
Study risk of bias assessment	11	Specify the methods used to assess risk of bias in the included studies, including details of the tool(s) used, how many reviewers assessed each study and whether they worked independently, and if applicable, details of automation tools used in the process.	2-3
Effect measures	12	Specify for each outcome the effect measure(s) (e.g. risk ratio, mean difference) used in the synthesis or presentation of results.	2-3
Synthesis methods	13a	Describe the processes used to decide which studies were eligible for each synthesis (e.g. tabulating the study intervention characteristics and comparing against the planned groups for each synthesis (item #5)).	2-3
	13b	Describe any methods required to prepare the data for presentation or synthesis, such as handling of missing summary statistics, or data conversions.	2-3
	13c	Describe any methods used to tabulate or visually display results of individual studies and syntheses.	2-3
	13d	Describe any methods used to synthesize results and provide a rationale for the choice(s). If meta-analysis was performed, describe the model(s), method(s) to identify the presence and extent of statistical heterogeneity, and software package(s) used.	2-3
	13e	Describe any methods used to explore possible causes of heterogeneity among study results (e.g. subgroup analysis, meta-regression).	2-3
	13f	Describe any sensitivity analyses conducted to assess robustness of the synthesized results.	2-3

Section and Topic	Item #	Checklist item	Location where item is reported
Reporting bias assessment	14	Describe any methods used to assess risk of bias due to missing results in a synthesis (arising from reporting biases).	2-3
Certainty assessment	15	Describe any methods used to assess certainty (or confidence) in the body of evidence for an outcome.	2-3
**RESULTS**			
Study selection	16a	Describe the results of the search and selection process, from the number of records identified in the search to the number of studies included in the review, ideally using a flow diagram.	3-11
	16b	Cite studies that might appear to meet the inclusion criteria, but which were excluded, and explain why they were excluded.	3-11
Study characteristics	17	Cite each included study and present its characteristics.	3-11
Risk of bias in studies	18	Present assessments of risk of bias for each included study.	3-11
Results of individual studies	19	For all outcomes, present, for each study: (a) summary statistics for each group (where appropriate) and (b) an effect estimate and its precision (e.g. confidence/credible interval), ideally using structured tables or plots.	3-11
Results of syntheses	20a	For each synthesis, briefly summarise the characteristics and risk of bias among contributing studies.	3-11
	20b	Present results of all statistical syntheses conducted. If meta-analysis was done, present for each the summary estimate and its precision (e.g. confidence/credible interval) and measures of statistical heterogeneity. If comparing groups, describe the direction of the effect.	3-11
	20c	Present results of all investigations of possible causes of heterogeneity among study results.	3-11
	20d	Present results of all sensitivity analyses conducted to assess the robustness of the synthesized results.	3-11
Reporting biases	21	Present assessments of risk of bias due to missing results (arising from reporting biases) for each synthesis assessed.	3-11
Certainty of evidence	22	Present assessments of certainty (or confidence) in the body of evidence for each outcome assessed.	3-11
**DISCUSSION**			
Discussion	23a	Provide a general interpretation of the results in the context of other evidence.	11-13
	23b	Discuss any limitations of the evidence included in the review.	11-13
	23c	Discuss any limitations of the review processes used.	11-13
	23d	Discuss implications of the results for practice, policy, and future research.	11-13
**OTHER INFORMATION**			
Registration and protocol	24a	Provide registration information for the review, including register name and registration number, or state that the review was not registered.	2-3
	24b	Indicate where the review protocol can be accessed, or state that a protocol was not prepared.	2-3
	24c	Describe and explain any amendments to information provided at registration or in the protocol.	2-3
Support	25	Describe sources of financial or non-financial support for the review, and the role of the funders or sponsors in the review.	13
Competing interests	26	Declare any competing interests of review authors.	13
Availability of data, code and other materials	27	Report which of the following are publicly available and where they can be found: template data collection forms; data extracted from included studies; data used for all analyses; analytic code; any other materials used in the review.	13

Supplementary Table S2. Search queries regarding the changes of Intraocular pressure before and after Laser-Assisted In Situ Keratomileusis (LASIK) and Photorefractive Keratectomy (PRK) Refractive Surgery.

Database	Query	Results
**Pubmed**	(LASIK OR femtoLASIK OR "Femtosecond Laser-Assisted in Situ Keratomileusis" OR "laser-assisted in situ keratomileusis" OR PRK OR "photorefractive keratectomy") AND ("Intraocular Pressure" OR IOP)	773
**Scopus**	TITLE-ABS-KEY ((LASIK OR femtoLASIK OR "Femtosecond Laser-Assisted in Situ Keratomileusis" OR "laser-assisted in situ keratomileusis" OR PRK OR "photorefractive keratectomy") AND ("Intraocular Pressure" OR IOP))	753
**Cochrane Library**	(LASIK OR femtoLASIK OR "Femtosecond Laser-Assisted in Situ Keratomileusis" OR "laser-assisted in situ keratomileusis" OR PRK OR "photorefractive keratectomy") AND ("Intraocular Pressure" OR IOP)	115
**ScienceDirect**	(femtoLASIK OR "Femtosecond Laser-Assisted in Situ Keratomileusis" OR "laser-assisted in situ keratomileusis" OR PRK OR "photorefractive keratectomy") AND ("Intraocular Pressure" OR IOP)	155

Supplementary Table S3. Quality assessment results with the Newcastle-Ottawa Quality Assessment Scale (NOS) tool.

Study	Design	Selection	Comparability	Outcome	Score	Quality
Younis et al. 2020	cohort	**	*	**	5/9	moderate
Sadigh et al. 2012	cohort	***	*	**	6/9	moderate
Faucher et al. 1997	cohort	***	*	**	6/9	moderate
Rosa et al. 2008	cohort	***	**	**	7/9	high
Salouti et al. 2021	cohort	**	*	**	5/9	moderate
Alonso-Munoz et al. 2002	cohort	***	**	***	8/9	high
Bao et al. 2019	cohort	***	*	**	6/9	moderate
Schipper et al. 2000	cohort	**	*	**	5/9	moderate
Shin et al. 2015	cohort	***	*	**	6/9	moderate
Lee et al. 2017	cohort	***	**	**	7/9	high
Hemida et al. 2020	cohort	***	**	**	7/9	high
Pepose et al. 2006	cohort	***	**	***	8/9	high
Cao et al. 2019	cohort	***	**	**	7/9	high
Munger et al. 2001	cohort	**	*	**	5/9	moderate
Aristeidou et al. 2011	cohort	**	*	**	5/9	moderate
Dou et al. 2015	cohort	**	*	*	4/9	moderate
Ang et al. 2022	cohort	***	*	**	6/9	moderate
Osman et al. 2016	cohort	***	**	**	7/9	high
Chen et al. 2008	cohort	***	*	**	6/9	moderate
Ortiz et al. 2007	cohort	**	*	**	5/9	moderate
Ryan et al 2011	cohort	**	*	**	5/9	moderate

Duba et al. 2003	cohort	**	*	*	4/9	moderate
Lam et al. 2010	cohort	**	*	*	4/9	moderate
Vakili et al. 2002	cohort	***	*	**	6/9	moderate
Chen et al. 2019	cohort	***	**	**	7/9	high
Refai et al. 2016	cohort	***	**	**	7/9	high
Sefat et al. 2015	cohort	***	*	**	6/9	moderate
Hamed-Azzam et al. 2012	cohort	**	*	**	5/9	moderate
Naruse et al. 2004	cohort	***	*	**	6/9	moderate
T Ang et al. 2022	cohort	***	**	**	7/9	high
Rosa et al. 1998	cohort	**	*	*	4/9	moderate
Gimeno et al. 2000	cohort	***	*	**	6/9	moderate
Helmy et al. 2020	cohort	***	**	**	7/9	high
Li et al. 2016	cohort	****	*	**	7/9	high
Humayun et al. 2023	cohort	***	*	**	6/9	moderate
Bernardo et al. 2014	cohort	***	**	***	8/9	high
Nabil et al. 2023	cohort	***	**	**	7/9	high
Iglesias et al. 2022	cohort	***	*	**	6/9	moderate
Cronemberger et al. 2009	cohort	****	*	**	7/9	high
Garzozi et al. 2001	cohort	**	*	**	5/9	moderate
Fournier et al. 1998	cohort	**	*	*	4/9	moderate
Cheng et al. 2005	cohort	**	*	**	5/9	moderate
Shemesh et al. 2012	cohort	***	**	**	7/9	high
Iglesias et al. 2020	cohort	***	**	***	8/9	high
Qazi et al. 2009	cohort	***	**	***	8/9	high
Shemesh et al. 2007	cohort	**	*	*	4/9	moderate
Park et al. 2000	cohort	**	*	**	5/9	moderate
Abbasoglu et al. 1998	cohort	**	*	*	4/9	moderate
Mardelli et al. 1997	cohort	*	*	*	3/9	low
Chihara et al. 2005	cohort	**	*	**	5/9	moderate
Shah et al. 2009	cohort	***	**	**	7/9	high
Ruangvaravate et al. 2005	cohort	**	*	**	5/9	moderate
Duch et al. 2001	cohort	**	*	**	5/9	moderate
Arimoto et al. 2001	cohort	***	*	**	6/9	moderate

Supplementary Table S4. Baseline corneal characteristics of the included studies

Study	Ablation depth (μm)	Baseline CCT (μm)	Follow-up duration (months)
Younis et al. 2020	NA	544.5 ± 27.2	1
Sadigh et al. 2012	90.7 ± 30.0	563.2 ± 35.1	1
Faucher et al. 1997	NA	553.7 ± 37.2	6
Rosa et al. 2008	NA	NA	3
Salouti et al. 2021	79.0 ± 27.0	537.0 ± 23.0	3
Alonso-Munoz et al. 2002	NA	NA	6
Bao et al. 2019	89.8 ± 29.9	550.7 ± 22.6	3
Schipper et al. 2000	NA	NA	3
Shin et al. 2015	85.0 ± 29.1	548.6 ± 27.2	1
Lee et al. 2017	92.8 ± 24.5	559.9 ± 27.8	6
Hemida et al. 2020	NA	564.6 ± 32.3	1
Pepose et al. 2006	NA	550.7 ± 34.3	1
Cao et al. 2019	NA	534.2 ± 28.9	3
Munger et al. 2001	NA	555.0 ± 3.0	6
Aristeidou et al. 2011	NA	554.8 ± 30.7	3
Dou et al. 2015	57.1 ± 15.3	532.0 ± 32.4	3
Ang et al. 2022	85.2 ± 24.3	552.3 ± 27.8	3
Osman et al. 2016	NA	530.4 ± 16.3	3
Chen et al. 2008	72.1 ± 28.8	549.9 ± 29.5	3
Ortiz et al. 2007	NA	560.0 ± 38.0	3
Ryan et al 2011	43.4 ± 14.9	538.4 ± 30.9	3
Duba et al. 2003	NA	555.0 ± 30.0	3
Lam et al. 2010	NA	544.9 ± 27.6	3
Vakili et al. 2002	NA	566 ± 44	6
Chen et al. 2019	91.90 ± 21.5	542.4 ± 21.4	3
Refai et al. 2016	72.3 ± 31.2	547.4 ± 24.1	3
Sefat et al. 2015	72.6 ± 18.3	556.2 ± 29.7	3
Hamed-Azzam et al. 2012	NA	560.3 ± 26.3	4
Naruse et al. 2004	NA	543.2 ± 32.5	3
T Ang et al. 2022	NA	550.0 ± 34.0	3
Rosa et al. 1998	NA	NA	6
Gimeno et al. 2000	NA	NA	6
Helmy et al. 2020	59.2 ± 22.0	546.9 ± 31.8	6
Li et al. 2016	91.1 ± 19.5	544.8 ± 28.3	6
Humayun et al. 2023	NA	540.9 ± 27.6	6
Bernardo et al. 2014	NA	NA	6
Nabil et al. 2023	61.3 ± 25.7	509.5 ± 30.0	6
Iglesias et al. 2022	70.5 ± 26.8	543.0 ± 27.0	3
Cronemberger et al. 2009	NA	533.0 ± 29.3	3
Garzozi et al. 2001	75.5 ± NA	537.6 ± 6.0	12

Fournier et al. 1998	76.6 ± 29.5	NA	3
Cheng et al. 2005	NA	NA	3
Shemesh et al. 2012	NA	562.3 ± 22.6	3
Iglesias et al. 2020	66.3 ± 26.2	549.9 ± 32.0	3
Qazi et al. 2009	94.8 ± 29.4	558.8 ± 27.9	6
Shemesh et al. 2007	NA	NA	3
Park et al. 2000	69.4 ± 25.1	598.8 ± 28.7	3
Abbasoglu et al. 1998	56.2 ± 16.7	537.0 ± 4.0	3
Mardelli et al. 1997	NA	551.0 ± 37.0	12
Chihara et al. 2005	66.1 ± 22.9	529.0 ± 32.0	3
Shah et al. 2009	NA	557.4 ± 36.3	3
Ruangvaravate et al. 2005	NA	523.7 + 24.0	3
Duch et al. 2001	77.1 ± NA	556.5 ± 31.8	3
Arimoto et al. 2001	NA	NA	3

Data are presented as mean ± standard deviation (SD). µm: micrometers; CCT: central corneal thickness; NA: not available/not reported.
